# Development of a large language model clinical variable extraction pipeline for the study of lymphedema

**DOI:** 10.1016/j.jvssci.2026.100448

**Published:** 2026-09-21

**Authors:** Andrea T. Fisher, Anoushka Lakshmi, Benjamin C. Liu, Jenny Woo, Richard Gaus, Akshay S. Chaudhari, Shipra Arya, Katherine Hekman

**Affiliations:** a Stanford Division of Vascular Surgery, Department of Surgery, Stanford University, Stanford, CA; b Stanford Center for Artificial Intelligence in Medicine and Imaging, Stanford University, Stanford, CA; c Stanford Department of Radiology, Stanford University, Stanford, CA; d Technical University of Munich, Munich, DE; e Stanford Department of Biomedical Data Science, Stanford University, Stanford, CA; f Stanford Cardiovascular Institute, Stanford, CA; g Weill Cancer Hub West, San Francisco, CA; h VA Palo Alto Healthcare System, Palo Alto, CA

**Keywords:** Artificial intelligence, Clinical data extraction, Large language models, Lymphedema

## Abstract

For many disease processes, clinical information is often captured in unstructured clinical notes that require manual data extraction for research. Large language models (LLMs) have the potential to automate the data extraction process, enabling study for diseases with fewer tabular metrics available. Here, we report the development and validation of an LLM-based clinical note data extraction approach for a retrospective cohort study of lymphedema etiologies and treatments. After using International Classification of Diseases, 10th Revision codes to identify potential lymphedema patients and text-matching to find potentially relevant clinical notes, an LLM was used to further filter notes, summarize for relevant content, and extract per-patient variables concerning etiology, treatments and efficacies, clinical stage, and wound status. Prompts were optimized for accuracy with an iterative review strategy, starting with zero-shot approaches and modified to few-shot approaches where necessary, validated by subset human review. A Protected Health Information-compliant Application Programming Interface version of OpenAI’s GPT-5 was used for all LLM queries. Human review of LLM-extracted final variables revealed high accuracy when compared against both an intermediate summarization step (99% accurate) and against original clinical notes (98% accurate) across 2858 patients/32,288 notes. LLMs are a valuable tool for conducting clinical research at scale using unstructured text data.

In the past year, frontier large language models (LLMs) such as OpenAI ChatGPT, Anthropic Claude Opus, and Google Gemini have demonstrated rapid advancements, allowing them to approach expert-level performance across a variety of tasks.^1^, ^2^, ^3^ There has been significant interest in using LLMs for medical domain tasks to broaden access to care, reduce physician workloads, and improve patient outcomes.^4^^,^^5^ LLMs are also a promising tool for medical research and have been used in diverse capacities from agentic discovery of novel therapeutic targets^6^ to systematic review of medical literature.^7^ In retrospective clinical research, relevant information such as clinical improvement or workup history is often stored in clinical notes and previously required manual review for variable extraction. However, LLMs have also demonstrated sufficient accuracy to replace or augment human labor for a variety of these chart review tasks.^8^, ^9^, ^10^, ^11^, ^12^

Lymphedema remains relatively understudied,^13^ perhaps partly because relevant information (such as etiologies considered, treatments tried, efficacy, and stage) is often captured in clinical notes rather than structured data fields. Since this information is labor-intensive to extract at scale, we devised and evaluated an LLM approach to clinical note data extraction for a retrospective cohort study of lymphedema etiologies and treatments. As general-purpose frontier models have begun to outperform smaller, clinically fine-tuned models on medical tasks,^9^^,^^14^, ^15^, ^16^ we elected to use a frontier model rather than a fine-tuned LLM. Although use of LLMs for clinical data extraction tasks has been validated previously,^17^^,^^18^ we are the first to describe LLM data extraction for lymphedema at scale. Our method uses a systematic approach of zero-shot and few-shot prompts, intermediate summarization, and manual subset validation. We provide a proof-of-concept for large-scale extraction of disease metrics traditionally captured in narrative form, using a frontier model in an iterative approach to achieve high output accuracy.

## Methods and validation

### Dataset

We queried our institution’s electronic health record (EHR) research database (Stanford STARR) for patients with an International Classification of Diseases, 10th Revision code for lymphedema (I89.0) between 2023 and 2025, identifying 6090 candidate charts with 2,455,234 total clinical notes. For each patient, all available EHR data were acquired, including demographic information and all clinical notes. Institutional Review Board (IRB) approval was obtained from our institution (Stanford University, IRB Reference No. 82854).

### LLM use and data privacy

Our institution offers access to a variety of frontier models approved for use with Protected Health Information (PHI; Stanford SecureGPT). The SecureGPT Application Programming was used to run all described LLM queries with a PHI-compliant version of OpenAI’s GPT-5.^19^ At the time this research was performed, investigators were not charged for use of this resource. Pricing has subsequently fluctuated depending on contracts between our institution and frontier laboratories offering these models. Data were stored and processed on a machine encrypted for use with PHI.

### Extraction approach design considerations

Design of the LLM prompt is key to ensuring the quality of the output data. Zero-shot prompts are defined as task requests provided without examples of the desired output. Few-shot prompts entail providing several examples of desired output in addition to the input data and task. We found that open-ended, zero-shot prompts worked well for filtering relevant notes and generating accurate summaries from raw clinical note text. We then evaluated zero-shot-only prompts to extract final variables from intermediate steps in LLM summarization (Supplementary Table I, online only). Review of variables extracted with a zero-shot strategy revealed that the LLM misunderstood what concepts constituted compressive wraps, surgery, trauma as etiology, and pneumatic pump device; few-shot/reworded prompts were necessary for these variables. Subsequently, performance was optimized in our study through a combination of zero-shot and few-shot prompting.

We next considered whether layers of summarization between original note text and variable extraction could introduce gradual distortions. However, previous extraction tasks using LLM clinical summaries have demonstrated advantages.^17^^,^^20^ This is consistent with findings from nonmedical LLM applications that more tokens—that is, the basic unit of text an LLM reads or writes, often a few characters—spent thinking, synthesizing, and verifying an answer will often yield better results.^21^^,^^22^ In our case, enforcing multiple passes (note-level summarization, patient-level summarization, final variable extraction) over the same information used more tokens to process the data, yet accuracy against the original source text remained high (as demonstrated in Table).

**Fig:**
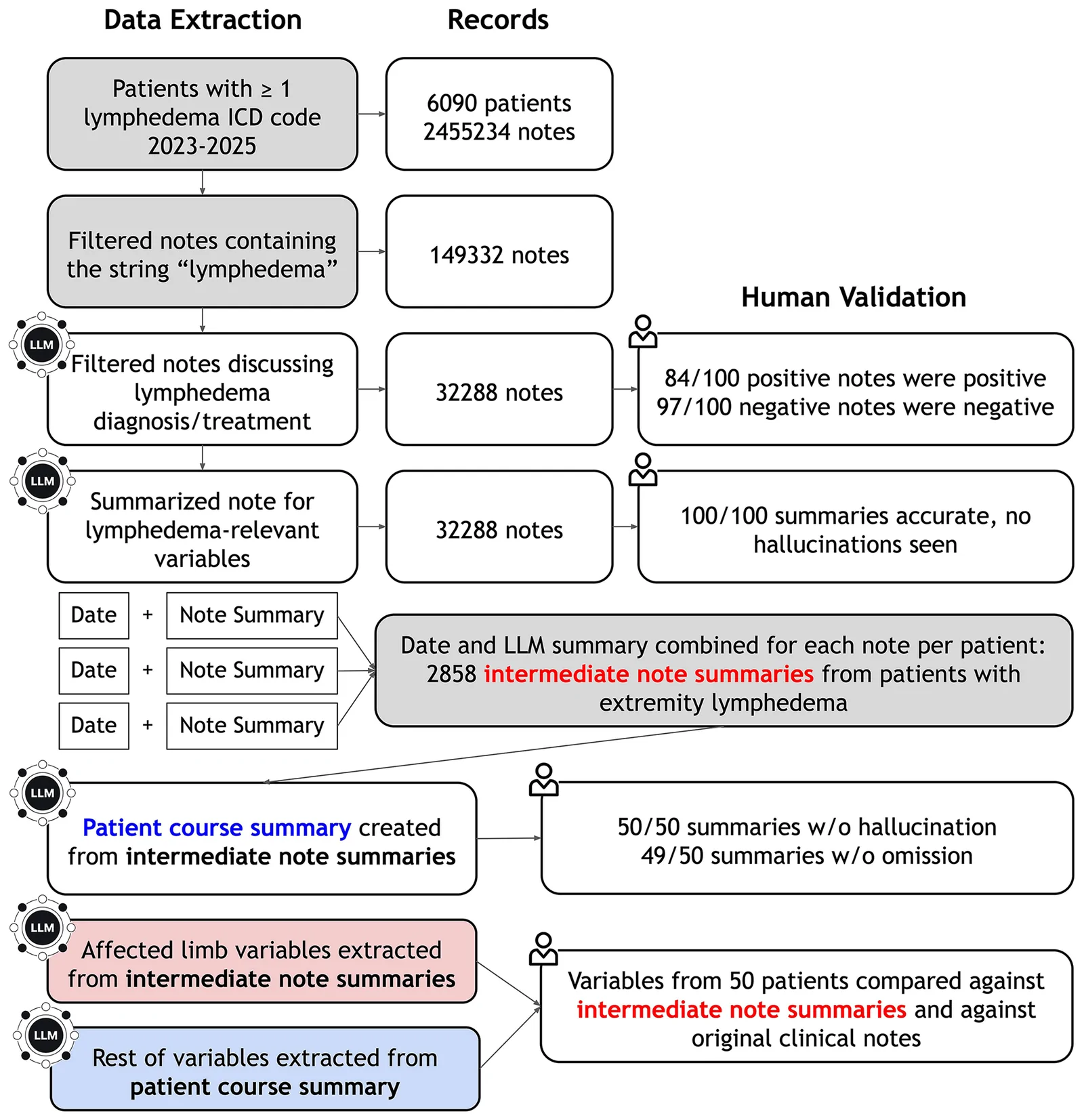
Data extraction and large language models (*LLM*) validation flowchart, from electronic health record (EHR) data acquisition to categorical variable extraction. For each LLM extraction step, a random sample of LLM outputs were validated by a human. *ICD*, International Classification of Diseases.

### Execution of clinical variable extraction

Variables of interest included those that were contained within EHR tabular data (such as sex, recent body mass index, and smoking status) and those contained within the text of clinical notes. Binary variables extracted from clinical notes were presence of lymphedema on right leg, left leg, right arm, left arm; etiologies including post-thrombotic, chronic venous reflux, congestive heart failure, congenital, cancer, trauma (not including surgical dissection as a cause of trauma), infectious; treatments including pneumatic pump, manual decongestive therapy, compressive wraps, surgical referral, surgery (including vascularized lymph node transfer, lymphaticovenous anastomosis, and venous procedures, but not including wound debridement); and outcomes including wound on lymphedema limb, wound improvement, and wound healing. Other outcome/disease progression variables were coded as categorical variables: initial and most recent lymphedema stage were collected as 0, 1, 2, 3, or unknown, and swelling improvement was graded as none, transient, or sustained.

To obtain categorical variables from clinical notes, we first filtered and summarized clinical notes for each patient (Fig). Each step performed by an LLM was validated via human review of a random subset of outputs. Validation was not blinded: for each validation step, LLM outputs were compared with the input text and deemed incorrect if they contradicted the input or omitted relevant information.

1. Text-matching was used to filter for only notes containing the string “lymphedema” (149,332 notes remaining).
2. An LLM was used to categorize each text-matched note as either containing or not containing discussion of a patient’s lymphedema diagnosis/treatment to exclude notes where lymphedema was imported in an automated diagnosis list and not otherwise discussed (32,288 notes remaining). Human validation of a random subset of included/excluded notes revealed that 84/100 included notes were indeed positive for lymphedema discussion, and 97/100 excluded notes did not contain any useful lymphedema information.
3. An LLM was used to generate a *note summary* of lymphedema-relevant details (such as note type, affected body part, underlying cause/etiology, current stage, treatments received, and treatment efficacy) from each lymphedema-relevant note. Of 100 summaries reviewed for fidelity to the original note, 100 were accurate with regards to summarized lymphedema details, and no hallucinations were seen.
4. For each patient, each note summary was appended with corresponding note date, and all *note summaries* with date were concatenated into a single set of *intermediate note summaries (1/patient).*
5. An LLM was used to generate a *patient course summary* from each patient’s set of *intermediate note summaries*, with particular attention to evidence for various etiologies, treatments tried, and change in lymphedema symptoms over time. Overall, 49/50 per-patient summaries were accurate. The inaccurate summary had an omission of relevant information in the patient course summary (one affected leg was not specifically mentioned). No hallucinations were seen at this step.

Then, we iterated through several extraction approaches to obtain the final binary and categorical variables, evaluating accuracy at each step. We first used a zero-shot method to extract all variables from the patient course summary (from step 5). We then retried the same zero-shot prompts to extract variables from the intermediate note summaries (step 4 product) instead of the patient course summary and compared outputs from both strategies on the same set of patients (Supplementary Table I, online only). Accuracy was generally high (>90%) for both approaches across all variables, with the exception of compressive wraps (78%) and pneumatic pump (82%). Close inspection of these errors led us to add few-shot and reworded prompts for some variables and adjust our extraction source for each variable.

For the final extraction strategy, we used zero-shot extraction of affected limb data from intermediate note summaries and a mix of zero- and few-shot extraction of the remaining variables from each patient course summary. Few-shot prompting was used for surgery and compressive wraps, and clarifying language was added for pneumatic pump, manual decongestive therapy, and trauma (Appendix, online only, step 6, prompt 2). Improved swelling was elicited as a categorical variable (eg, none, transient, and sustained). For lymphedema stage, the LLM was prompted to either give a numerical stage (0-3) or 9 if it was unsure (Appendix, online only, step 6, prompt 3).

Evaluations of final-stage LLM outputs were performed by authors A.L., B.L., and J.W and reviewed by author A.T.F. Reviewers compared LLM extracted variables against each patient’s intermediate note summaries with flag incorrect outputs. For a random set of 50 patients (a different set from the previous validation steps), variable extraction accuracy was determined by comparing each variable against the content of the corresponding intermediate note summaries. For a random subset of 25 of these patients, the accuracy of each variable was recalculated against the original clinical notes that remained for a given patient after filtering out notes that did not contain the word “lymphedema,” with high accuracy (>90%) throughout (Table).

**Table:** Summary of variables and accuracies

Categories	Extraction sources	Variables	Accuracy against intermediate note summaries	Accuracy from original clinical notes
Limb	Intermediate note summaries	Right leg	49/50 (98)	24/25 (96)
		Left leg	49/50 (98)	24/25 (96)
		Right arm	50/50 (100)	25/25 (100)
		Left arm	50/50 (100)	25/25 (100)
Etiologies	Patient course summary	Post-thrombotic	50/50 (100)	24/25 (96)
		Chronic venous insufficiency	50/50 (100)	25/25 (100)
		Congestive heart failure	49/50 (98)	25/25 (100)
		Congenital	50/50 (100)	25/25 (100)
		Cancer	50/50 (100)	25/25 (100)
		Trauma	50/50 (100)	25/25 (100)
		Infectious	49/50 (98)	24/25 (96)
Treatments	Patient course summary	Pneumatic pump	50/50 (100)	23/25 (92)
		Manual decongestive therapy	49/50 (98)	24/25 (96)
		Compressive wraps	50/50 (100)	25/25 (100)
		Surgical referral	49/50 (98)	25/25 (100)
		Surgery	49/50 (98)	24/25 (96)
Outcomes	Patient course summary	Improved swelling (none, transient, and sustained)	48/50 (96)	24/25 (96)
		Presenting stage (0, 1, 2, 3, and unknown)	49/50 (98)	24/25 (96)
		Recent stage (0, 1, 2, 3, and unknown)	47/50 (94)	23/25 (92)
		Wound on lymphedema limb	50/50 (100)	25/25 (100)
		Wound improved	50/50 (100)	25/25 (100)
		Wound healed	50/50 (100)	25/25 (100)

Accuracy was computed against intermediate, large language models (LLM)-generated note summaries for a random subset of 50 patients, and against the original clinical notes for a random subset of 25 patients.

Categorical data are shown as number (%).

Extracting lymphedema stage presented additional barriers, in part due to inconsistent explicit documentation of the clinical stage by providers. We noted that the LLM would occasionally add a stage designation based on physical examination/clinical severity descriptions, despite being given the option to omit the stage from its answer if it was unsure, so we validated the stage information in greater detail. Note summaries often copied forward relevant physical examination descriptions from the source text, allowing stage addition even from intermediate steps. From clinical note to note summary (step 3), only 13 of 100 reviewed clinical notes contained explicit stage information, and the LLM added a stage number in 9/100 summaries, all of which were correct based on comparison with the source clinical note. From intermediate note summaries (step 4) to patient course summaries (step 5), 33 of 50 intermediate note summaries had at least one explicit stage designation, and stage information was added in 7/50 patient course summaries with 100% accuracy. Finally, from patient course summaries to extracted variables, the presenting stage was present in 22/50 and the recent stage was present in 19/50; stage data was added in 11 instances and was inaccurate in 3/11 cases. (Supplementary Fig, online only). At the final extraction step when the LLM was asked to designate the presenting and recent stage, staging errors were introduced even though the LLM was allowed to respond “unsure.” Given the low proportion of original notes with stage information explicitly stated (13%), we found it beneficial for the LLM to add occasional stage interpretations, despite noise introduced in the last step.

All prompts used in the final extraction pipeline are available in the Appendix (online only), along with example input/output pairs. Cohort variable data is available in Supplementary Table II (online only).

## Conclusions

Here, we present a novel iterative approach that combines multiple prompting strategies and intermediate summary steps to achieve high accuracy on a large lymphedema dataset. Over 6000 patients with over 2 million notes were analyzed in a high-throughput fashion, with >90% accuracy in the extraction of clinical variables, as confirmed by manual validation. This approach is a valuable addition to clinical research methods for disease processes that have historically been captured in narrative form with limited structured data.

Our approach nonetheless has several limitations. Because accuracy varies with the prompt and the variable being extracted, rigorous adaptation of our technique to other tasks and diseases will require manual review of a subset of LLM outputs to ensure high-quality extraction. Undetected systematic errors can jeopardize the validity of downstream analysis: For example, had we not specifically evaluated the accuracy of pneumatic pump and compressive wrap variables at the intermediate stage, associations between these treatments and outcome metrics would be less reliable. Complete elimination of error is often not possible, but knowledge of accuracy (and recall/precision for variables with rare prevalence)^23^ aids interpretation of downstream statistical associations.

Although we employ human review of LLM outputs, we do not compare independently obtained human-extracted variables against LLM outputs. We opted against this approach both because of feasibility constraints and because we wished to identify and characterize LLM mistakes rather than demonstrate that LLM data extraction is equivalent to human performance. Numerous prior publications validate the general use of even older, less-advanced LLMs for data extraction across medical domains, including cognitive impairment severity and social determinants of health from clinical notes, hepatocellular carcinoma tumor characteristics from radiology reports, and lung cancer characteristics from pathology reports.^8^^,^^9^^,^^17^^,^^18^^,^^24^ Finally, LLM-introduced noise is a risk to downstream analysis tasks whenever LLMs are used for intermediate data extraction steps. However, manual chart review at a large scale, as described, is frequently not feasible without the use of an automated tool. Despite these modest accuracy considerations, optimized LLM variable extraction allows performance of retrospective clinical research at an unprecedented scale.

LLMs can be effectively used as a high-accuracy tool for large-scale clinical research. Prompt iteration in the development of a pipeline helps to empirically maximize accuracy, and well-designed summarization steps can allow an LLM to sequentially distill knowledge without introducing hallucinations or widespread distortions. Evaluation of per-variable LLM extraction accuracy via subset review and careful inventory of systematic patterns of error are essential steps in implementation of this technique for valid downstream analysis. Our method can be easily adapted to extract variables from clinical notes for the study of other pathologies at scale.

## Funding

A.T.F receives funding through T32HL166155 from the 10.13039/100000002National Institutes of Health, Stanford Cardiovascular Institute, Computational Medicine in the Heart: Integrated Training Program, which supported this work. B.L. receives funding through the Stanford Cardiovascular Institute Boring Trust. A.S.C. receives funding through ARPA-H contracts AY2AX000045 and 1AYSAX0000024-01, which supported this work. S.A. is supported by grants from 10.13039/100000968American Heart Association (25TPA1479692) and 10.13039/100000002National Institutes of Health/National Heart Lung and Blood Institute (UG3HL181184, 1R01HL166305-01A) and Veterans Affairs Office of Research and Development (I01HX003215). K.H. is supported by funding through Department of Veterans Affairs, Career Development Award, IK2 BX006275.

## Disclosures

A.T.F., A.L., B.C.L., J.W., R.G., A.S.C., S.A., and K.H. have no conflicts of interest.

## Figures and Tables

**Supplementary Fig:**
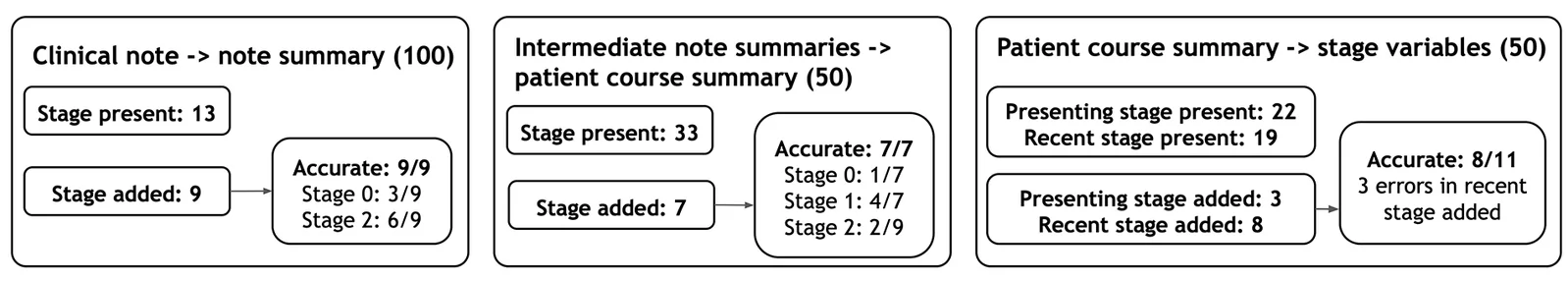


## Appendix (online only)

Supplementary Tables I and II (online only)
