# Supplementary material for: Development of a large language model clinical variable extraction pipeline for the study of lymphedema
Source: JVS Vasc Sci. 2026 Sep 21;7:100448. doi: 10.1016/j.jvssci.2026.100448 (PMC13692102; doi:10.1016/j.jvssci.2026.100448)
Supplement: Supplementary file 1 [file mmc1.docx]

| **Category** | **Variable** | **Patient course summary** | **Intermediate note summaries** |
| --- | --- | --- | --- |
| Limb | Right leg | 48/50 (96%) | 49/50 (98%) |
|  | Left leg | 50/50 (100%) | 50/50 (100%) |
|  | Right arm | 50/50 (100%) | 50/50 (100%) |
|  | Left arm | 50/50 (100%) | 50/50 (100%) |
| Etiologies | Post-thrombotic | 50/50 (100%) | 50/50 (100%) |
|  | Chronic venous insufficiency | 50/50 (100%) | 49/50 (98%) |
|  | Congestive heart failure | 50/50 (100%) | 48/50 (96%) |
|  | Congenital | 50/50 (100%) | 50/50 (100%) |
|  | Cancer | 50/50 (100%) | 49/50 (98%) |
|  | Trauma | 49/50 (98%) | 48/50 (96%) |
|  | Infectious | 50/50 (100%) | 48/50 (96%) |
| Treatments | Pneumatic pump | 45/50 (90%) | 41/50 (82%) |
|  | Manual decongestive therapy | 49/50 (98%) | 48/50 (96%) |
|  | Compressive wraps | 39/50 (78%) | 45/50 (90%) |
|  | Surgical referral | 50/50 (100%) | 45/50 (90%) |
|  | Surgery | 48/50 (96%) | 47/50 (94%) |
| Outcomes | Improved swelling | 50/50 (100%) | 47/50 (94%) |
|  | Presenting stage | 50/50 (100%) | 46/50 (92%) |
|  | Recent stage | 47/50 (94%) | 44/50 (88%) |
|  | Wound on lymphedema limb | 50/50 (100%) | 47/50 (94%) |
|  | Wound improved | 49/50 (98%) | 46/50 (92%) |
|  | Wound healed | 49/50 (98%) | 45/50 (90%) |

**Supplemental Table S1:** Variable extraction accuracy from intermediate iterations of prompt strategies. Accuracy was computed against intermediate note summaries, regardless of extraction source, for a random subset of 50 patients. For presenting and recent stage, stage was considered inaccurate either if the given stage disagreed with information in the intermediate note summaries, or if stage provided was “unsure” when a numeric stage was available.

| **Category** | **Variable** | **N (%) / Avg (SD)** |
| --- | --- | --- |
| Demographics | Female sex | 2162 (76%) |
|  | BMI | 31.4 (9.9) |
|  | Ever smoker | 776 (27%) |
| Limb | Right leg | 1707 (60%) |
|  | Left leg | 1833 (64%) |
|  | Right arm | 555 (19%) |
|  | Left arm | 595 (21%) |
| Etiologies | Post-thrombotic | 219 (8%) |
|  | Chronic venous insufficiency | 818 (29%) |
|  | Congestive heart failure | 109 (4%) |
|  | Congenital | 280 (10%) |
|  | Cancer | 1307 (46%) |
|  | Trauma | 241 (8%) |
|  | Infectious | 76 (3%) |
| Treatments | Pneumatic pump | 977 (34%) |
|  | Manual decongestive therapy | 1404 (49%) |
|  | Compressive wraps | 2330 (82%) |
|  | Surgical referral | 728 (25%) |
|  | Surgery | 436 (15%) |
| Outcomes | Improved swelling: none | 1040 (36%) |
|  | Improved swelling: transient | 897 (31%) |
|  | Improved swelling: sustained | 921 (32%) |
|  | Presenting stage: 0 | 170 (6%) |
|  | Presenting stage: 1 | 493 (17%) |
|  | Presenting stage: 2 | 754 (26%) |
|  | Presenting stage: 3 | 129 (5%) |
|  | Presenting stage: unknown | 1312 (46%) |
|  | Recent stage: 0 | 116 (4%) |
|  | Recent stage: 1 | 401 (14%) |
|  | Recent stage: 2 | 1017 (36%) |
|  | Recent stage: 3 | 253 (9%) |
|  | Recent stage: unknown | 1071 (37%) |
|  | Wound on lymphedema limb | 598 (21%) |
|  | Wound improved | 366 (61%) |
|  | Wound healed | 235 (39%) |

**Table S2:** Demographics, etiologies, treatments, and outcomes present in the study cohort. Percentages for wound improvement and wound healing are calculated from patients who developed a wound.
