# Supplementary material for: Development of a large language model clinical variable extraction pipeline for the study of lymphedema
Source: JVS Vasc Sci. 2026 Sep 21;7:100448. doi: 10.1016/j.jvssci.2026.100448 (PMC13692102; doi:10.1016/j.jvssci.2026.100448)
Supplement: Supplementary file 2 [file mmc2.docx]

**Appendix**

LLM system prompts for each step of data extraction are listed here, along with example input and output pairs. Each system prompt is paired with a user prompt that includes the input text. Inputs for content below were fabricated to avoid release of PHI; no actual patient data is included here.

***Step 2: Categorization of notes as positive or negative for lymphedema discussion***

**Prompt:** “You are a medical research assistant extracting information from clinical notes. you are deciding if each clinical note is focused on the diagnosis or management of a patient's lymphedema. if lymphedema diagnosis/management is the focus, please respond with a 1. if lymphedema management/diagnosis is not discussed, or if lymphedema is not the focus of the note, please respond with a 0. do not include any additional words or content in your response.”

**Input:**

Discharge Summary Hospitalization Details: Attending Physician: Priya Deshmukh, MD Attending Physician Contact Info: Office 650-555-2301; Pager 650-555-2379 Additional discharging provider: Samuel Ortiz, NP Discharging service: Medicine Admission Date: 05/28/2026 Discharge Date: 06/01/2026 Identifier: Patient ID: 58M with history of Hodgkin lymphoma in remission s/p right axillary lymph node dissection and radiation (2010) complicated by chronic right upper extremity (RUE) lymphedema, hypertension, CKD3a (baseline Cr ~1.2–1.3), hypothyroidism, gout, and hyperlipidemia, presenting with acute RUE erythema and pain consistent with cellulitis superimposed on chronic lymphedema. Reason for Hospitalization: right upper extremity cellulitis in the setting of chronic lymphedema To-Do List PCP: FYI: Completed 48 hours IV cefazolin with clinical improvement; discharge on cephalexin 500 mg QID to complete 10-day course [ ] Reassess blood pressure on new regimen (losartan continued; HCTZ discontinued; started amlodipine 5 mg daily) [ ] Recheck BMP in 1–2 weeks to monitor potassium and creatinine (CKD3a, on ARB) [ ] Consider prophylactic penicillin VK if recurrent cellulitis episodes (e.g., ≥2/year) Reinforce lymphedema self-management: no BP/venipuncture in RUE, daily emollient, prompt treatment of skin breaks Referral to outpatient lymphedema therapy placed; verify compression garment fitting and MLD training follow-up Brief HPI - Per Admission H&P: As per admission H&P: "Patient reports sudden onset of redness, warmth, and swelling of the right arm starting 24 hours prior to presentation with subjective fevers and chills. He has longstanding right arm lymphedema after axillary lymph node removal and radiation in 2010. Typically managed with a compression sleeve and manual lymph drainage, but he has not worn his sleeve consistently in the past month due to summer heat. No recent trauma or insect bites recalled. Denies shortness of breath or chest pain. No prior DVTs. No known MRSA colonization." ED Course: On arrival T 38.6°C, HR 112, BP 156/88, RR 18, SpO2 98% RA. Exam showed diffuse erythema and tenderness over forearm and upper arm with chronic nonpitting edema; no crepitus or bullae. Labs with WBC 14.2, lactate 2.1. Two sets of blood cultures obtained. Started empiric IV cefazolin; vancomycin deferred given low MRSA risk and nonpurulent cellulitis. Upper extremity venous duplex obtained to exclude DVT—negative. Admitted for IV antibiotics and lymphedema care. Hospital Course and Discharge Plan, by Issue: Brief Summary: Admitted 05/28/2026 for acute nonpurulent cellulitis of the RUE on a background of chronic secondary lymphedema after prior axillary dissection and radiation for Hodgkin lymphoma. Treated with IV cefazolin for 48 hours with resolution of fever and down-trending leukocytosis, then transitioned to oral cephalexin to complete a 10-day course. Blood cultures remained negative. Lymphedema therapy evaluated and initiated short-stretch compression bandaging after erythema improved; patient trained in skin care and precautions (no venipuncture/BP in RUE). Hypertension regimen adjusted (discontinued HCTZ; started amlodipine) given CKD and gout. Discharged home with outpatient lymphedema therapy and PCP follow-up. Hospital Course by Problem: Acute nonpurulent cellulitis, right upper extremity, on chronic lymphedema Presentation with fever, leukocytosis, diffuse erythema/warmth without purulence. No signs of necrotizing infection; lactate normalized with fluids. MRSA nares negative; blood cultures x2 no growth to date (NGTD at 72 hours). Responded to IV cefazolin 2 g q8h x 48 hours; erythema margins receded and pain improved. Transitioned to cephalexin 500 mg PO QID to complete 10-day total course (including inpatient days). Education on early signs of recurrence and need for prompt antibiotics provided. Cephalexin 500 mg PO QID to complete course through 06/07/2026 Elevation, gentle active ROM; acetaminophen for pain/fever If ≥2 episodes/year, consider prophylaxis (penicillin VK 250 mg BID) after ID/PCP discussion Secondary lymphedema, right upper extremity (post-axillary dissection/radiation) Chronic RUE lymphedema with intermittent sleeve use preceding cellulitis. Lymphedema therapy (OT) consulted: initiated decongestive therapy plan including manual lymph drainage (MLD), short-stretch multi-layer bandaging after acute inflammatory phase improved, and measurement for custom compression sleeve/glove (daytime) and nighttime garment. Skin care reinforced (daily emollient, antiseptic wash as needed, prompt treatment of tinea/dermatitis). Outpatient lymphedema therapy referral placed (6–8 sessions for MLD, bandaging, garment training) Prescription provided for custom daytime class 2 compression sleeve with gauntlet; nighttime quilted garment Strict limb precautions: avoid venipuncture/BP in RUE; protect from trauma/heat Hypertension Home regimen included losartan 50 mg daily and hydrochlorothiazide 25 mg daily. Given CKD3a and gout, and mild prerenal azotemia on presentation, HCTZ discontinued. Initiated amlodipine 5 mg daily with good BP control (SBP 120s–130s) by discharge. Continue losartan 50 mg daily Start amlodipine 5 mg daily Discontinue HCTZ PCP to monitor BP and BMP in 1–2 weeks Chronic kidney disease, stage 3a (baseline Cr ~1.2–1.3 mg/dL; eGFR ~55–60) Admission Cr 1.34 improved to 1.24 with fluids and avoidance of nephrotoxins. No proteinuria assessment during admission. Avoid NSAIDs; renally dose medications BMP recheck in 1–2 weeks after ARB/CCB regimen adjustment Hypothyroidism Clinically euthyroid; TSH 2.6 this admission. Continue levothyroxine 100 mcg daily; PCP to monitor TSH annually Gout No acute flare. Uric acid not checked this admission.

Continue allopurinol 100 mg daily; avoid thiazide diuretic Hyperlipidemia Continue atorvastatin 20 mg nightly Discharge Exam: General Appearance: Comfortable, no acute distress Eyes: Sclera anicteric ENT: Oropharynx clear, moist mucous membranes Neck: Supple, no JVD Lungs: Clear to auscultation bilaterally, normal effort Cardiac: Regular rate and rhythm; no murmurs, rubs, or gallops Abdomen: Soft, nontender, nondistended Extremities: RUE with chronic nonpitting edema; faint residual erythema of mid-forearm without warmth or tenderness; improved range of motion. No fluctuance. LUE and lower extremities without edema. Neuro: Alert and oriented x3; no focal deficits Skin: Intact; RUE skin with chronic thickening; no open wounds; no tinea noted Psychiatric: Appropriate affect Laboratory Studies: CBC: Recent Labs 05/30/26 06:10 05/31/26 05:58 06/01/26 05:47 WBC 11.2 8.9 7.6 HGB 13.8 13.4 13.6 HCT 41.5 40.2 40.8 PLT 232 246 255 Electrolytes: Recent Labs 05/30/26 06:10 05/31/26 05:58 06/01/26 05:47 NA 139 140 138 K 4.2 4.1 4.3 CL 103 104 102 CO2 25 26 25 BUN 24 21 20 CR 1.30 1.26 1.24 CA 9.1 9.2 9.1 Glucose: Recent Labs 05/30/26 06:10 05/31/26 05:58 06/01/26 05:47 GLU 112 106 103 Coagulation Studies: No results for PT, PTT, INR in the last 72 hours.

LFTs: Recent Labs 05/30/26 06:10 05/31/26 05:58 06/01/26 05:47 TBIL 0.5 0.4 0.4 AST 19 17 18 ALT 21 20 22 ALKP 74 70 71 ALB 3.7 3.6 3.6 Microbiology: Blood cultures (2/2) collected 05/28/26: No growth at 72 hours MRSA nasal PCR: Not detected Imaging: Right Upper Extremity Venous Duplex Ultrasound Result Date: 05/29/2026 CLINICAL HISTORY: 58-year-old male with acute right arm swelling and erythema; rule out DVT. TECHNIQUE: Real-time grayscale and color Doppler ultrasound of the right internal jugular, subclavian, axillary, brachial, basilic, and cephalic veins. FINDINGS: Normal flow and compressibility of all evaluated deep venous segments. No intraluminal thrombus identified. Diffuse subcutaneous tissue thickening and increased echogenicity consistent with edema/lymphedema. IMPRESSION: No evidence of deep venous thrombosis in the right upper extremity. Subcutaneous edema consistent with lymphedema/cellulitis. Author: H. Nguyen, MD Attending: H. Nguyen, MD Procedures: None Discharge Medications: What to do with your medications TAKE these medications cephalexin 500 mg capsule Instructions: Take 1 capsule (500 mg) by mouth four times daily for 8 more days to complete a 10-day course (last dose 06/07/2026) acetaminophen 500 mg tablet Instructions: Take 1 tablet (500 mg) by mouth every 6 hours as needed for pain or fever (max 3,000 mg/day) amlodipine 5 mg tablet Instructions: Take 1 tablet (5 mg) by mouth daily losartan 50 mg tablet Instructions: Take 1 tablet (50 mg) by mouth daily levothyroxine 100 mcg tablet Instructions: Take 1 tablet (100 mcg) by mouth every morning on an empty stomach atorvastatin 20 mg tablet Instructions: Take 1 tablet (20 mg) by mouth nightly allopurinol 100 mg tablet Instructions: Take 1 tablet (100 mg) by mouth daily plain petrolatum ointment Instructions: Apply a thin layer to RUE daily after bathing to maintain skin barrier STOP TAKING these medications hydrochlorothiazide 25 mg tablet ibuprofen and other NSAIDs (avoid due to CKD) Where to Get Your Medications Prescription Printed - may take to any pharmacy Bring a paper prescription for each of these medications: cephalexin 500 mg capsule amlodipine 5 mg tablet petrolatum ointment Existing home medications to continue as listed above. Discharge Orders: Discharge Procedure Orders Referral to Lymphedema Rehabilitation (Occupational Therapy) Referral Priority: Routine Referral Type: Consult, Treat Referral Reason: Specialty Services Requested (MLD, bandaging, garment fitting) Number of Visits Requested: 6 Durable Medical Equipment/Compression Order Comments: Measure and fit for custom daytime class 2 RUE compression sleeve with gauntlet; nighttime quilted garment. Educate on donning/doffing and wear schedule. Discharge Diet Order Specific Question Answer Comments Diet Heart healthy (low sodium) 2 g Na PT/OT/SLP Evaluation Order Specific Question Answer Comments Rehab OT evaluation per facility Outpatient lymphedema therapy Rehab PT evaluation per facility Home exercise program for shoulder/elbow ROM Interagency Referral to Home Health (if available) Skilled nursing for first-week check on antibiotic adherence and skin assessment Vital signs and RUE circumference monitoring twice weekly x 2 weeks, then PRN Wound/Skin Care Order Comments: No open wounds requiring dressings Daily gentle cleansing; apply emollient to RUE after bathing Begin short-stretch multi-layer bandaging once cellulitis resolved per OT guidance Limb precautions: No venipuncture, IVs, or blood pressure measurements in RUE Non Invasive Positive Pressure Ventilation Not applicable Teaching Physician Attestation I saw and examined the patient and discussed management with the NP. I reviewed the NP’s note and agree with the documented findings and plan of care, with the addition and/or exception of the items documented below: History: 58-year-old man with chronic secondary RUE lymphedema presenting with acute nonpurulent cellulitis; improved on IV cefazolin. Physical Examination Findings: Afebrile; improving RUE erythema; persistent nonpitting edema without tenderness; no fluctuance. Impression and Plan: Cellulitis superimposed on lymphedema, responding to therapy—transition to oral cephalexin to complete 10 days; counsel on recurrence prevention and limb precautions. Chronic lymphedema—resume compression/MLD with OT; fit for custom garments. HTN—continue losartan, start amlodipine; stop HCTZ. CKD3a—avoid NSAIDs; BMP recheck in 1–2 weeks. Continue levothyroxine, allopurinol, and atorvastatin. Outpatient lymphedema therapy scheduled. Time spent: I personally spent a total of 30 minutes on the floor/unit and at bedside, including 18 minutes counseling and coordinating care regarding cellulitis management, lymphedema precautions and therapy plan, hypertension regimen changes, and CKD medication safety.

**Output:** 1

***Step 3: Note summarization for lymphedema-relevant variables***

**Prompt:**

“You are a medical research assistant extracting information on lymphedema from clinical notes. if any of this content is present in the note, please extract and summarize the following elements: note type, affected body part, underlying cause or etiology of lymphedema (cancer, venous insufficiency, venous reflux, post-DVT, trauma, congenital, infectious disease, or other cause), stage at presentation, treatments received, and treatment efficacy.”

**Input:**

Discharge Summary Hospitalization Details: Attending Physician: Priya Deshmukh, MD Attending Physician Contact Info: Office 650-555-2301; Pager 650-555-2379 Additional discharging provider: Samuel Ortiz, NP Discharging service: Medicine Admission Date: 05/28/2026 Discharge Date: 06/01/2026 Identifier: Patient ID: 58M with history of Hodgkin lymphoma in remission s/p right axillary lymph node dissection and radiation (2010) complicated by chronic right upper extremity (RUE) lymphedema, hypertension, CKD3a (baseline Cr ~1.2–1.3), hypothyroidism, gout, and hyperlipidemia, presenting with acute RUE erythema and pain consistent with cellulitis superimposed on chronic lymphedema. Reason for Hospitalization: right upper extremity cellulitis in the setting of chronic lymphedema To-Do List PCP: FYI: Completed 48 hours IV cefazolin with clinical improvement; discharge on cephalexin 500 mg QID to complete 10-day course [ ] Reassess blood pressure on new regimen (losartan continued; HCTZ discontinued; started amlodipine 5 mg daily) [ ] Recheck BMP in 1–2 weeks to monitor potassium and creatinine (CKD3a, on ARB) [ ] Consider prophylactic penicillin VK if recurrent cellulitis episodes (e.g., ≥2/year) Reinforce lymphedema self-management: no BP/venipuncture in RUE, daily emollient, prompt treatment of skin breaks Referral to outpatient lymphedema therapy placed; verify compression garment fitting and MLD training follow-up Brief HPI - Per Admission H&P: As per admission H&P: "Patient reports sudden onset of redness, warmth, and swelling of the right arm starting 24 hours prior to presentation with subjective fevers and chills. He has longstanding right arm lymphedema after axillary lymph node removal and radiation in 2010. Typically managed with a compression sleeve and manual lymph drainage, but he has not worn his sleeve consistently in the past month due to summer heat. No recent trauma or insect bites recalled. Denies shortness of breath or chest pain. No prior DVTs. No known MRSA colonization." ED Course: On arrival T 38.6°C, HR 112, BP 156/88, RR 18, SpO2 98% RA. Exam showed diffuse erythema and tenderness over forearm and upper arm with chronic nonpitting edema; no crepitus or bullae. Labs with WBC 14.2, lactate 2.1. Two sets of blood cultures obtained. Started empiric IV cefazolin; vancomycin deferred given low MRSA risk and nonpurulent cellulitis. Upper extremity venous duplex obtained to exclude DVT—negative. Admitted for IV antibiotics and lymphedema care. Hospital Course and Discharge Plan, by Issue: Brief Summary: Admitted 05/28/2026 for acute nonpurulent cellulitis of the RUE on a background of chronic secondary lymphedema after prior axillary dissection and radiation for Hodgkin lymphoma. Treated with IV cefazolin for 48 hours with resolution of fever and down-trending leukocytosis, then transitioned to oral cephalexin to complete a 10-day course. Blood cultures remained negative. Lymphedema therapy evaluated and initiated short-stretch compression bandaging after erythema improved; patient trained in skin care and precautions (no venipuncture/BP in RUE). Hypertension regimen adjusted (discontinued HCTZ; started amlodipine) given CKD and gout. Discharged home with outpatient lymphedema therapy and PCP follow-up. Hospital Course by Problem: Acute nonpurulent cellulitis, right upper extremity, on chronic lymphedema Presentation with fever, leukocytosis, diffuse erythema/warmth without purulence. No signs of necrotizing infection; lactate normalized with fluids. MRSA nares negative; blood cultures x2 no growth to date (NGTD at 72 hours). Responded to IV cefazolin 2 g q8h x 48 hours; erythema margins receded and pain improved. Transitioned to cephalexin 500 mg PO QID to complete 10-day total course (including inpatient days). Education on early signs of recurrence and need for prompt antibiotics provided. Cephalexin 500 mg PO QID to complete course through 06/07/2026 Elevation, gentle active ROM; acetaminophen for pain/fever If ≥2 episodes/year, consider prophylaxis (penicillin VK 250 mg BID) after ID/PCP discussion Secondary lymphedema, right upper extremity (post-axillary dissection/radiation) Chronic RUE lymphedema with intermittent sleeve use preceding cellulitis. Lymphedema therapy (OT) consulted: initiated decongestive therapy plan including manual lymph drainage (MLD), short-stretch multi-layer bandaging after acute inflammatory phase improved, and measurement for custom compression sleeve/glove (daytime) and nighttime garment. Skin care reinforced (daily emollient, antiseptic wash as needed, prompt treatment of tinea/dermatitis). Outpatient lymphedema therapy referral placed (6–8 sessions for MLD, bandaging, garment training) Prescription provided for custom daytime class 2 compression sleeve with gauntlet; nighttime quilted garment Strict limb precautions: avoid venipuncture/BP in RUE; protect from trauma/heat Hypertension Home regimen included losartan 50 mg daily and hydrochlorothiazide 25 mg daily. Given CKD3a and gout, and mild prerenal azotemia on presentation, HCTZ discontinued. Initiated amlodipine 5 mg daily with good BP control (SBP 120s–130s) by discharge. Continue losartan 50 mg daily Start amlodipine 5 mg daily Discontinue HCTZ PCP to monitor BP and BMP in 1–2 weeks Chronic kidney disease, stage 3a (baseline Cr ~1.2–1.3 mg/dL; eGFR ~55–60) Admission Cr 1.34 improved to 1.24 with fluids and avoidance of nephrotoxins. No proteinuria assessment during admission. Avoid NSAIDs; renally dose medications BMP recheck in 1–2 weeks after ARB/CCB regimen adjustment Hypothyroidism Clinically euthyroid; TSH 2.6 this admission. Continue levothyroxine 100 mcg daily; PCP to monitor TSH annually Gout No acute flare. Uric acid not checked this admission.

Continue allopurinol 100 mg daily; avoid thiazide diuretic Hyperlipidemia Continue atorvastatin 20 mg nightly Discharge Exam: General Appearance: Comfortable, no acute distress Eyes: Sclera anicteric ENT: Oropharynx clear, moist mucous membranes Neck: Supple, no JVD Lungs: Clear to auscultation bilaterally, normal effort Cardiac: Regular rate and rhythm; no murmurs, rubs, or gallops Abdomen: Soft, nontender, nondistended Extremities: RUE with chronic nonpitting edema; faint residual erythema of mid-forearm without warmth or tenderness; improved range of motion. No fluctuance. LUE and lower extremities without edema. Neuro: Alert and oriented x3; no focal deficits Skin: Intact; RUE skin with chronic thickening; no open wounds; no tinea noted Psychiatric: Appropriate affect Laboratory Studies: CBC: Recent Labs 05/30/26 06:10 05/31/26 05:58 06/01/26 05:47 WBC 11.2 8.9 7.6 HGB 13.8 13.4 13.6 HCT 41.5 40.2 40.8 PLT 232 246 255 Electrolytes: Recent Labs 05/30/26 06:10 05/31/26 05:58 06/01/26 05:47 NA 139 140 138 K 4.2 4.1 4.3 CL 103 104 102 CO2 25 26 25 BUN 24 21 20 CR 1.30 1.26 1.24 CA 9.1 9.2 9.1 Glucose: Recent Labs 05/30/26 06:10 05/31/26 05:58 06/01/26 05:47 GLU 112 106 103 Coagulation Studies: No results for PT, PTT, INR in the last 72 hours.

LFTs: Recent Labs 05/30/26 06:10 05/31/26 05:58 06/01/26 05:47 TBIL 0.5 0.4 0.4 AST 19 17 18 ALT 21 20 22 ALKP 74 70 71 ALB 3.7 3.6 3.6 Microbiology: Blood cultures (2/2) collected 05/28/26: No growth at 72 hours MRSA nasal PCR: Not detected Imaging: Right Upper Extremity Venous Duplex Ultrasound Result Date: 05/29/2026 CLINICAL HISTORY: 58-year-old male with acute right arm swelling and erythema; rule out DVT. TECHNIQUE: Real-time grayscale and color Doppler ultrasound of the right internal jugular, subclavian, axillary, brachial, basilic, and cephalic veins. FINDINGS: Normal flow and compressibility of all evaluated deep venous segments. No intraluminal thrombus identified. Diffuse subcutaneous tissue thickening and increased echogenicity consistent with edema/lymphedema. IMPRESSION: No evidence of deep venous thrombosis in the right upper extremity. Subcutaneous edema consistent with lymphedema/cellulitis. Author: H. Nguyen, MD Attending: H. Nguyen, MD Procedures: None Discharge Medications: What to do with your medications TAKE these medications cephalexin 500 mg capsule Instructions: Take 1 capsule (500 mg) by mouth four times daily for 8 more days to complete a 10-day course (last dose 06/07/2026) acetaminophen 500 mg tablet Instructions: Take 1 tablet (500 mg) by mouth every 6 hours as needed for pain or fever (max 3,000 mg/day) amlodipine 5 mg tablet Instructions: Take 1 tablet (5 mg) by mouth daily losartan 50 mg tablet Instructions: Take 1 tablet (50 mg) by mouth daily levothyroxine 100 mcg tablet Instructions: Take 1 tablet (100 mcg) by mouth every morning on an empty stomach atorvastatin 20 mg tablet Instructions: Take 1 tablet (20 mg) by mouth nightly allopurinol 100 mg tablet Instructions: Take 1 tablet (100 mg) by mouth daily plain petrolatum ointment Instructions: Apply a thin layer to RUE daily after bathing to maintain skin barrier STOP TAKING these medications hydrochlorothiazide 25 mg tablet ibuprofen and other NSAIDs (avoid due to CKD) Where to Get Your Medications Prescription Printed - may take to any pharmacy Bring a paper prescription for each of these medications: cephalexin 500 mg capsule amlodipine 5 mg tablet petrolatum ointment Existing home medications to continue as listed above. Discharge Orders: Discharge Procedure Orders Referral to Lymphedema Rehabilitation (Occupational Therapy) Referral Priority: Routine Referral Type: Consult, Treat Referral Reason: Specialty Services Requested (MLD, bandaging, garment fitting) Number of Visits Requested: 6 Durable Medical Equipment/Compression Order Comments: Measure and fit for custom daytime class 2 RUE compression sleeve with gauntlet; nighttime quilted garment. Educate on donning/doffing and wear schedule. Discharge Diet Order Specific Question Answer Comments Diet Heart healthy (low sodium) 2 g Na PT/OT/SLP Evaluation Order Specific Question Answer Comments Rehab OT evaluation per facility Outpatient lymphedema therapy Rehab PT evaluation per facility Home exercise program for shoulder/elbow ROM Interagency Referral to Home Health (if available) Skilled nursing for first-week check on antibiotic adherence and skin assessment Vital signs and RUE circumference monitoring twice weekly x 2 weeks, then PRN Wound/Skin Care Order Comments: No open wounds requiring dressings Daily gentle cleansing; apply emollient to RUE after bathing Begin short-stretch multi-layer bandaging once cellulitis resolved per OT guidance Limb precautions: No venipuncture, IVs, or blood pressure measurements in RUE Non Invasive Positive Pressure Ventilation Not applicable Teaching Physician Attestation I saw and examined the patient and discussed management with the NP. I reviewed the NP’s note and agree with the documented findings and plan of care, with the addition and/or exception of the items documented below: History: 58-year-old man with chronic secondary RUE lymphedema presenting with acute nonpurulent cellulitis; improved on IV cefazolin. Physical Examination Findings: Afebrile; improving RUE erythema; persistent nonpitting edema without tenderness; no fluctuance. Impression and Plan: Cellulitis superimposed on lymphedema, responding to therapy—transition to oral cephalexin to complete 10 days; counsel on recurrence prevention and limb precautions. Chronic lymphedema—resume compression/MLD with OT; fit for custom garments. HTN—continue losartan, start amlodipine; stop HCTZ. CKD3a—avoid NSAIDs; BMP recheck in 1–2 weeks. Continue levothyroxine, allopurinol, and atorvastatin. Outpatient lymphedema therapy scheduled. Time spent: I personally spent a total of 30 minutes on the floor/unit and at bedside, including 18 minutes counseling and coordinating care regarding cellulitis management, lymphedema precautions and therapy plan, hypertension regimen changes, and CKD medication safety.

**Output:**

- Note type: Discharge Summary

- Affected body part: Right upper extremity (arm; forearm and upper arm)

- Underlying cause/etiology of lymphedema: Cancer-related (secondary to Hodgkin lymphoma treatment with right axillary lymph node dissection and radiation in 2010)

- Stage at presentation: Not explicitly documented. Exam describes chronic nonpitting edema with chronic skin thickening, consistent with at least ISL Stage II, but stage not stated in the note.

- Treatments received:

- Cellulitis management: IV cefazolin 2 g q8h for 48 hours, then cephalexin 500 mg PO QID to complete a 10-day total course; elevation; acetaminophen for pain/fever.

- Lymphedema management: Decongestive therapy plan with manual lymph drainage (MLD); short-stretch multi-layer compression bandaging initiated after erythema improved; measurement/prescription for custom daytime class 2 compression sleeve with gauntlet and a nighttime quilted garment; skin care (daily emollient; limb precautions including no venipuncture/BP in RUE); referral to outpatient lymphedema therapy (6–8 sessions) for MLD, bandaging, and garment training; gentle active ROM.

- Treatment efficacy:

- Cellulitis: Improved—fever resolved, leukocytosis down-trended (WBC 14.2 → 7.6), erythema receded, pain improved; blood cultures remained negative.

- Lymphedema: Management initiated and compression planned; persistent chronic nonpitting edema at discharge; no quantitative limb volume reduction documented during admission.

***Step 5: Patient course summary generation from intermediate note summaries***

**Prompt:**

**“**You are a medical research assistant summarizing each patient's lymphedema diagnosis and treatment from summaries of clinical data. please use the following summary to answer these questions, if possible: What etiologies of lymphedema were considered and what evidence exists for each etiology? What was the stage at initial presentation and how did it change over time? What treatments were trialed, and what was the efficacy of each one?**”**

**Input:**

Date: 1/3/2025

- Note type: Discharge Summary

- Affected body part: Right upper extremity (arm; forearm and upper arm)

- Underlying cause/etiology of lymphedema: Cancer-related (secondary to Hodgkin lymphoma treatment with right axillary lymph node dissection and radiation in 2010)

- Stage at presentation: Not explicitly documented. Exam describes chronic nonpitting edema with chronic skin thickening, consistent with at least ISL Stage II, but stage not stated in the note.

- Treatments received:

- Cellulitis management: IV cefazolin 2 g q8h for 48 hours, then cephalexin 500 mg PO QID to complete a 10-day total course; elevation; acetaminophen for pain/fever.

- Lymphedema management: Decongestive therapy plan with manual lymph drainage (MLD); short-stretch multi-layer compression bandaging initiated after erythema improved; measurement/prescription for custom daytime class 2 compression sleeve with gauntlet and a nighttime quilted garment; skin care (daily emollient; limb precautions including no venipuncture/BP in RUE); referral to outpatient lymphedema therapy (6–8 sessions) for MLD, bandaging, and garment training; gentle active ROM.

- Treatment efficacy:

- Cellulitis: Improved—fever resolved, leukocytosis down-trended (WBC 14.2 → 7.6), erythema receded, pain improved; blood cultures remained negative.

- Lymphedema: Management initiated and compression planned; persistent chronic nonpitting edema at discharge; no quantitative limb volume reduction documented during admission.

Date: 2/5/2026

- Note type: Outpatient lymphedema follow-up clinic note

- Affected body part: Right upper extremity (hand to proximal upper arm)

- Underlying cause/etiology of lymphedema: Cancer-related (secondary to Hodgkin lymphoma treatment with right axillary lymph node dissection and radiation)

- Stage at presentation: ISL Stage II, stable (nonpitting with reduced fibrosis compared to prior; no active infection)

- Treatments received:

- Completed initial 6-week course of complete decongestive therapy (CDT) in mid 2025 (MLD, short-stretch bandaging, exercise, skin care), transitioned to maintenance phase

- Daytime custom class 2 compression sleeve with gauntlet worn 8–10 hours/day; nighttime quilted garment; garments refitted/replaced at 6 months

- Self-MLD 10–15 minutes daily and structured home exercise program (diaphragmatic breathing; hand/wrist/elbow ROM; shoulder abduction with scapular setting)

- Added home pneumatic compression device (sequential pump) January 2026: 30 minutes daily, low-pressure regimen

- Ongoing skin care (daily gentle cleanse; bland emollient; nail care) and limb precautions (no BP/venipuncture/IVs in RUE; avoid trauma/heat; elevation when resting)

- No antibiotic prophylaxis initiated (no recurrent cellulitis episodes)

- Treatment efficacy:

- Limb volume trend: Baseline (01/2025) RUE ~4,050 mL vs LUE ~3,250 mL (excess 800 mL; ~24.6%); at 12 months (01/2026) RUE ~3,650 mL vs LUE ~3,300 mL (excess 350 mL; ~10.6%) → ~56% reduction in excess volume

- Circumference improvements: mid-forearm −2.0 cm; upper arm −1.8 cm from baseline

- Functional gains: shoulder abduction improved from ~150° to ~170°; reports less heaviness and improved endurance for daily tasks (typing, light lifting)

- Skin status: intact without erythema or wounds; occasional mild garment-related irritation resolved with refitting and emollients

- Infections: 0 cellulitis recurrences in the past year; no ED visits or hospitalizations related to lymphedema

**Output:**

Etiologies considered and supporting evidence:

- Cancer-related (secondary lymphedema): Supported by prior Hodgkin lymphoma treatment with right axillary lymph node dissection and radiation in 2010, followed by chronic right arm swelling (nonpitting edema, skin thickening). This is explicitly documented as the underlying cause in both notes.

- Other etiologies: Not supported in the provided summaries. Venous thrombosis was evaluated and excluded by duplex ultrasound (no DVT). There is no documentation suggesting venous insufficiency/reflux, trauma, congenital, or primary infectious causes of lymphedema; cellulitis was present as a superimposed infection rather than the etiology of the chronic lymphedema.

Stage at initial presentation and change over time:

- Initial (1/3/2025): Stage not explicitly stated; exam findings of chronic nonpitting edema with skin thickening are consistent with at least ISL Stage II.

- Follow-up (~12–13 months later, 2/5/2026): Documented as ISL Stage II, stable, with reduced fibrosis compared to prior and no active infection.

- Overall change: Stage remained ISL Stage II; tissue quality improved (reduced fibrosis), with objective reduction in excess limb volume and improved function.

Treatments trialed and efficacy:

- Acute cellulitis treatment (1/2025): IV cefazolin 2 g q8h for 48 hours → oral cephalexin 500 mg QID to complete 10 days; elevation; acetaminophen. Efficacy: Fever resolved, leukocytosis declined (WBC 14.2 → 7.6), erythema receded, pain improved, blood cultures negative; no cellulitis recurrences in the subsequent year, so no prophylactic antibiotics were started.

- Complete decongestive therapy (CDT) (mid 2025): Course included manual lymph drainage (MLD), short-stretch multi-layer bandaging, therapeutic exercises, and skin care. Efficacy: Marked improvement over 12 months—excess limb volume decreased from ~800 mL (~24.6%) to ~350 mL (~10.6%), a ~56% reduction in excess volume; forearm circumference −2.0 cm and upper arm −1.8 cm; functional gains (shoulder abduction ~150° → ~170°, improved endurance; less heaviness).

- Compression therapy: Custom daytime class 2 sleeve with gauntlet (8–10 h/day) and nighttime quilted garment, with refitting at 6 months. Efficacy: Contributed to sustained volume reduction and symptom control; minor garment-related skin irritation resolved with refitting and emollients.

- Self-management/maintenance: Daily self-MLD (10–15 minutes), structured home exercise program, ongoing skin care and limb precautions. Efficacy: Supported maintenance of volume reduction and prevention of infections (0 cellulitis episodes over the year).

- Home pneumatic compression device (added 1/2026): 30 minutes daily, low-pressure regimen. Efficacy: Implemented late in the year; considered part of the maintenance strategy accompanying observed volume reduction by the 12-month assessment, though isolated effect size is not separately quantified in the notes.

***Step 6: Variable extraction***

**Prompt 1:** Affected limb from intermediate note summaries

“you are a medical research assistant extracting variables about each patient's lymphedema diagnosis and treatment from their timestamped summarized clinical notes. please use the following note summaries to provide variables as either 1 (yes) or 0 (no), comma separated within brackets. presence of lymphedema on right leg, on left leg, on right arm, on left arm. Do not add any additional text. Answer should appear like [1, 0, 1, 0] with 4 total elements (this example shows someone with right leg and right arm lymphedema).”

**Input 1:**

Date: 1/3/2025

- Note type: Discharge Summary

- Affected body part: Right upper extremity (arm; forearm and upper arm)

- Underlying cause/etiology of lymphedema: Cancer-related (secondary to Hodgkin lymphoma treatment with right axillary lymph node dissection and radiation in 2010)

- Stage at presentation: Not explicitly documented. Exam describes chronic nonpitting edema with chronic skin thickening, consistent with at least ISL Stage II, but stage not stated in the note.

- Treatments received:

- Cellulitis management: IV cefazolin 2 g q8h for 48 hours, then cephalexin 500 mg PO QID to complete a 10-day total course; elevation; acetaminophen for pain/fever.

- Lymphedema management: Decongestive therapy plan with manual lymph drainage (MLD); short-stretch multi-layer compression bandaging initiated after erythema improved; measurement/prescription for custom daytime class 2 compression sleeve with gauntlet and a nighttime quilted garment; skin care (daily emollient; limb precautions including no venipuncture/BP in RUE); referral to outpatient lymphedema therapy (6–8 sessions) for MLD, bandaging, and garment training; gentle active ROM.

- Treatment efficacy:

- Cellulitis: Improved—fever resolved, leukocytosis down-trended (WBC 14.2 → 7.6), erythema receded, pain improved; blood cultures remained negative.

- Lymphedema: Management initiated and compression planned; persistent chronic nonpitting edema at discharge; no quantitative limb volume reduction documented during admission.

Date: 2/5/2026

- Note type: Outpatient lymphedema follow-up clinic note

- Affected body part: Right upper extremity (hand to proximal upper arm)

- Underlying cause/etiology of lymphedema: Cancer-related (secondary to Hodgkin lymphoma treatment with right axillary lymph node dissection and radiation)

- Stage at presentation: ISL Stage II, stable (nonpitting with reduced fibrosis compared to prior; no active infection)

- Treatments received:

- Completed initial 6-week course of complete decongestive therapy (CDT) in mid 2025 (MLD, short-stretch bandaging, exercise, skin care), transitioned to maintenance phase

- Daytime custom class 2 compression sleeve with gauntlet worn 8–10 hours/day; nighttime quilted garment; garments refitted/replaced at 6 months

- Self-MLD 10–15 minutes daily and structured home exercise program (diaphragmatic breathing; hand/wrist/elbow ROM; shoulder abduction with scapular setting)

- Added home pneumatic compression device (sequential pump) January 2026: 30 minutes daily, low-pressure regimen

- Ongoing skin care (daily gentle cleanse; bland emollient; nail care) and limb precautions (no BP/venipuncture/IVs in RUE; avoid trauma/heat; elevation when resting)

- No antibiotic prophylaxis initiated (no recurrent cellulitis episodes)

- Treatment efficacy:

- Limb volume trend: Baseline (01/2025) RUE ~4,050 mL vs LUE ~3,250 mL (excess 800 mL; ~24.6%); at 12 months (01/2026) RUE ~3,650 mL vs LUE ~3,300 mL (excess 350 mL; ~10.6%) → ~56% reduction in excess volume

- Circumference improvements: mid-forearm −2.0 cm; upper arm −1.8 cm from baseline

- Functional gains: shoulder abduction improved from ~150° to ~170°; reports less heaviness and improved endurance for daily tasks (typing, light lifting)

- Skin status: intact without erythema or wounds; occasional mild garment-related irritation resolved with refitting and emollients

- Infections: 0 cellulitis recurrences in the past year; no ED visits or hospitalizations related to lymphedema

**Output 1:** [0, 0, 1, 0]

**Prompt 2:** Binary variables from patient course summary

“You are a medical research assistant extracting variables from a summary of each patient's lymphedema diagnosis and treatment. please use the following summary to provide variables as either 1 (yes) or 0 (no), comma separated within brackets. post-thrombotic/DVT etiology, chronic venous insufficiency etiology, CHF etiology, congenital etiology, infectious etiology (NOT including cellulitis/subsequent infections after lymphedema diagnosis), cancer etiology, traumatic injury etiology (not including trauma from prior surgery), whether patient was referred for surgical intervention for lymphedema, whether patient received surgery for lymphedema (including VLNT or vascularized lymph node transfer, LVA or lymphaticovenous anastomosis, and any vein procedures or stents for venous reflux or obstruction, but NOT including wound debridements), use of pneumatic pump (only code as a 1 if a patient has been confirmed to have used a pneumatic pump, not just received a prescription for one. Pneumatic pump is a mechanical device that squeezes fluid out of the tissues. Do not include “ankle pump” or “muscle pump”, which are actually names of exercises a patient can perform to increase fluid return from the legs), manual decongestive therapy (MLD or manual lymphatic drainage should be coded the same as MDT, manual decongestive therapy. This is where a practitioner performs massage therapy that moves fluid out of the affected area. CDT or complete decongestive therapy includes MDT and compressive wraps), compressive wraps (includes compressive sleeves, compression stockings, compression socks, ace wrap, compressive bandages such as Unna boot), presence of wound on lymphedema limb, whether or not it healed (if no wound had developed, code as 0) and whether wound has improved. Do not add any additional text. Answer should appear like [1, 0, 1, 0, 1, 0, 0, 0, 1, 0, 0, 0, 0, 0, 0] with 15 total elements.”

**Input 2:**

Etiologies considered and supporting evidence:

- Cancer-related (secondary lymphedema): Supported by prior Hodgkin lymphoma treatment with right axillary lymph node dissection and radiation in 2010, followed by chronic right arm swelling (nonpitting edema, skin thickening). This is explicitly documented as the underlying cause in both notes.

- Other etiologies: Not supported in the provided summaries. Venous thrombosis was evaluated and excluded by duplex ultrasound (no DVT). There is no documentation suggesting venous insufficiency/reflux, trauma, congenital, or primary infectious causes of lymphedema; cellulitis was present as a superimposed infection rather than the etiology of the chronic lymphedema.

Stage at initial presentation and change over time:

- Initial (1/3/2025): Stage not explicitly stated; exam findings of chronic nonpitting edema with skin thickening are consistent with at least ISL Stage II.

- Follow-up (~12–13 months later, 2/5/2026): Documented as ISL Stage II, stable, with reduced fibrosis compared to prior and no active infection.

- Overall change: Stage remained ISL Stage II; tissue quality improved (reduced fibrosis), with objective reduction in excess limb volume and improved function.

Treatments trialed and efficacy:

- Acute cellulitis treatment (1/2025): IV cefazolin 2 g q8h for 48 hours → oral cephalexin 500 mg QID to complete 10 days; elevation; acetaminophen. Efficacy: Fever resolved, leukocytosis declined (WBC 14.2 → 7.6), erythema receded, pain improved, blood cultures negative; no cellulitis recurrences in the subsequent year, so no prophylactic antibiotics were started.

- Complete decongestive therapy (CDT) (mid 2025): Course included manual lymph drainage (MLD), short-stretch multi-layer bandaging, therapeutic exercises, and skin care. Efficacy: Marked improvement over 12 months—excess limb volume decreased from ~800 mL (~24.6%) to ~350 mL (~10.6%), a ~56% reduction in excess volume; forearm circumference −2.0 cm and upper arm −1.8 cm; functional gains (shoulder abduction ~150° → ~170°, improved endurance; less heaviness).

- Compression therapy: Custom daytime class 2 sleeve with gauntlet (8–10 h/day) and nighttime quilted garment, with refitting at 6 months. Efficacy: Contributed to sustained volume reduction and symptom control; minor garment-related skin irritation resolved with refitting and emollients.

- Self-management/maintenance: Daily self-MLD (10–15 minutes), structured home exercise program, ongoing skin care and limb precautions. Efficacy: Supported maintenance of volume reduction and prevention of infections (0 cellulitis episodes over the year).

- Home pneumatic compression device (added 1/2026): 30 minutes daily, low-pressure regimen. Efficacy: Implemented late in the year; considered part of the maintenance strategy accompanying observed volume reduction by the 12-month assessment, though isolated effect size is not separately quantified in the notes.

**Output 2:** [0, 0, 0, 0, 0, 1, 0, 0, 0, 1, 1, 1, 0, 0, 0]

**Prompt 3:** Categorical variable extraction from patient course summary

“You are a medical research assistant extracting variables from a summary of each patient's lymphedema diagnosis and treatment. please use the following summary to provide integer values corresponding to: lymphedema stage at initial presentation (0, 1, 2, or 3) followed by most recent lymphedema stage (0, 1, 2, or 3). If you are unable to determine lymphedema stage, give it a value of 9. Finally, provide an integer value corresponding to improved swelling in response to treatment: 0 = no significant swelling improvement. 1 = Transient improvement, may at one point have had improved swelling, but doesn’t persist from visit to visit. 2 = Sustained improvement, swelling improvement persists over multiple visits. Do not add any additional text. Answers should appear in brackets, comma separated, like [1, 2, 0].”

**Input 3:**

Etiologies considered and supporting evidence:

- Cancer-related (secondary lymphedema): Supported by prior Hodgkin lymphoma treatment with right axillary lymph node dissection and radiation in 2010, followed by chronic right arm swelling (nonpitting edema, skin thickening). This is explicitly documented as the underlying cause in both notes.

- Other etiologies: Not supported in the provided summaries. Venous thrombosis was evaluated and excluded by duplex ultrasound (no DVT). There is no documentation suggesting venous insufficiency/reflux, trauma, congenital, or primary infectious causes of lymphedema; cellulitis was present as a superimposed infection rather than the etiology of the chronic lymphedema.

Stage at initial presentation and change over time:

- Initial (1/3/2025): Stage not explicitly stated; exam findings of chronic nonpitting edema with skin thickening are consistent with at least ISL Stage II.

- Follow-up (~12–13 months later, 2/5/2026): Documented as ISL Stage II, stable, with reduced fibrosis compared to prior and no active infection.

- Overall change: Stage remained ISL Stage II; tissue quality improved (reduced fibrosis), with objective reduction in excess limb volume and improved function.

Treatments trialed and efficacy:

- Acute cellulitis treatment (1/2025): IV cefazolin 2 g q8h for 48 hours → oral cephalexin 500 mg QID to complete 10 days; elevation; acetaminophen. Efficacy: Fever resolved, leukocytosis declined (WBC 14.2 → 7.6), erythema receded, pain improved, blood cultures negative; no cellulitis recurrences in the subsequent year, so no prophylactic antibiotics were started.

- Complete decongestive therapy (CDT) (mid 2025): Course included manual lymph drainage (MLD), short-stretch multi-layer bandaging, therapeutic exercises, and skin care. Efficacy: Marked improvement over 12 months—excess limb volume decreased from ~800 mL (~24.6%) to ~350 mL (~10.6%), a ~56% reduction in excess volume; forearm circumference −2.0 cm and upper arm −1.8 cm; functional gains (shoulder abduction ~150° → ~170°, improved endurance; less heaviness).

- Compression therapy: Custom daytime class 2 sleeve with gauntlet (8–10 h/day) and nighttime quilted garment, with refitting at 6 months. Efficacy: Contributed to sustained volume reduction and symptom control; minor garment-related skin irritation resolved with refitting and emollients.

- Self-management/maintenance: Daily self-MLD (10–15 minutes), structured home exercise program, ongoing skin care and limb precautions. Efficacy: Supported maintenance of volume reduction and prevention of infections (0 cellulitis episodes over the year).

- Home pneumatic compression device (added 1/2026): 30 minutes daily, low-pressure regimen. Efficacy: Implemented late in the year; considered part of the maintenance strategy accompanying observed volume reduction by the 12-month assessment, though isolated effect size is not separately quantified in the notes.

**Output 3:** [2, 2, 2]
